# Does historical land use affect the regional distribution of fleshy-fruited woody plants?

**DOI:** 10.1371/journal.pone.0225791

**Published:** 2019-12-05

**Authors:** Matilda Arnell, Sara A. O. Cousins, Ove Eriksson

**Affiliations:** 1 Department of Ecology, Environment and Plant Sciences, Stockholm University, Stockholm, Sweden; 2 Biogeography and Geomatics, Department of Physical Geography, Stockholm University, Stockholm, Sweden; Institute of Botany of the Czech Academy of Sciences, CZECH REPUBLIC

## Abstract

Species richness and composition of current vegetation may reflect historical land use. We develop and examine the hypothesis that regional distribution and richness of fleshy-fruited woody plants, a group sharing life-form and dispersal system, reflect historical land use in open or semi-open habitats. Historical land use was based on maps from around the year 1900 for two regions in Sweden, and field data was gathered from surveys made in these regions. Species richness was positively related to historical land use indicated as open habitat in 1900. In one of the regions, five out of nine examined species were positively related to historical land use (with historical effect R^2^ ranging between 0.03 and 0.22). In the other region, we found a weaker positive relationship with historical land use in two out of nine examined species (R^2^ 0.01 and 0.02). We conclude that current occurrence and richness of fleshy-fruited woody species is partly a legacy of historical land use, and that regions may vary in this respect. Based on a comparison between the two regions examined here, we discuss some potential causes behind this variation.

## Introduction

Human land use has profoundly transformed vegetation globally [1–3] affecting species distributions and species diversity. These effects can be long lasting and persist after land use practices have ceased or changed. It is generally acknowledged that legacies of historical land use on current vegetation are common [4–7]. Such legacies have been found in tropical forests [8], temperate forests and woodland [5,9,10] and in temperate grasslands [11–14]. Historical effects on current vegetation are interesting in their own right, for instance to understand how past land use has formed regional species pools in anthropogenic landscapes [15]. Several authors also stress that knowledge of historical effects may be important for understanding how current anthropogenic impact may shape future vegetation [6,7].

The effects of land use on species diversity may have historical roots extending back many centuries or even millennia. This is the case for European semi-natural grasslands historically used for hay-making and grazing [16–19]. Human settlement and land use increased the area and stabilized open habitats in space and time, which would have led to an accumulation of species [15]. This explains why the large species pool associated with semi-natural grasslands is present at most sites [15,20]. Today, many of these habitats are abandoned and this is associated with species loss [21].

While the effects of historical land use on species richness of forbs and grasses in semi-natural grasslands have been extensively studied [12–14,19,22], few studies have focused on other groups of plants. In this study, we ask the question whether historical land use may have had a similar effect on the richness and distribution of fleshy-fruited woody plants. These are trees and shrubs producing fruits such as berries and drupes, or fruit-like fleshy structures such as the female cones in junipers, making them attractive as food for animals, which thereby assist in seed dispersal. In temperate regions fleshy-fruited species are mainly dispersed by birds. We have two underlying reasons for expecting historical effects of land use on fleshy-fruited woody plants.

The hypothesis was based on a combination of effects, the human-mediated changes in landscape structure [15], the response of frugivores to such changes [23–25] and the intentional favouring of these species in managed habitats [26].

Our first reason concerns historical land use in combination with the life-history traits of this group of species: their habitat requirements, dispersal and regional population dynamics. In Scandinavia, large areas were historically used as meadows [18,19]. These meadows were part of infields [27–29], i.e. enclosed areas close to settlements also including arable fields, where grazing was either prevented or controlled (e.g. after hay harvest). Meadows were essential, as the production of hay and leaf-hay was used as winter fodder for livestock whose manure was used to fertilize permanent fields. Outside the infields and surrounding the farms or villages there were extensive areas (outlying land) used for grazing and for other natural resources [18]. This system of organizing land as infields and outlying land has its roots in the Iron Age, about two millennia BP [29]. Despite periods of expansion and abandonment, and technological innovation, it was basically maintained in Scandinavia until the modernization of agriculture and forestry during the 19^th^ and 20^th^ century [19].

Fleshy-fruited woody plants are often found in forest edge habitats, or in semi-open woodland [10,30,31]. These species would thus have been unintentionally favoured by the increased area and spatio-temporal stabilization of open and semi-open habitats similarly to grassland species by a decrease in local extinction rates and by increased dispersal and immigration rates [15]. This initial accumulation of fleshy-fruited species would have been reinforced by a generally positive relationship between fruit removal and seed deposition by frugivores and the abundance and diversity of fleshy-fruited species [23,32,33] creating an indirect link between human land use and dispersal of fleshy-fruited trees and shrubs in the landscape promoting an aggregation of species [24]. Such local assemblages of many species of fleshy-fruited species have been described as ‘orchards’ [25].

Secondly, fleshy-fruited woody plants may have been intentionally favoured on meadows close to settlements. Based on a detailed survey of the flora in wooded meadows in Åland archipelago in the Baltic Sea was published 1916 [34]. Wooded meadows in this landscape were meadows managed for production of leaf-hay (leaves and twigs from pollarded trees) and hay [10,35]. In 1916, these meadows were still managed and had been managed for at least several centuries [36]. Thirty different wooded meadows were surveyed, located on different islands in the Åland archipelago. Of all woody species recorded (44 in all), 27 (61%) were fleshy-fruited, and among these, 18 (76%) were present in more than 50% of the meadows (S1 Fig). Fleshy-fruited woody species were not the most important tree species for the production of leaf-hay [26], but they would have been kept intentionally because they provided fruits (e.g. *Malus* spp., *Prunus* spp., *Sorbus* spp.) [10,37] and important construction material used for wood-work [26]. The intentional favouring of fleshy-fruited woody plants for food and construction materials would likely have been practiced all over southern Scandinavia. Fleshy-fruited woody species would thus have accumulated both on infields as well as in the semi-open habitats on the border between open habitats and the outlying land. These distribution patterns would be manifested at a landscape scale, compared to the corresponding accumulation of grassland species, which is detected at small spatial scales [38].

Based on these considerations, we hypothesize that when land use increased the area of open and semi-open habitats and stabilized them in space and time [29], this resulted in an accumulation of fleshy-fruited woody species which would have been intentionally favoured by people managing the land. This would have been a continuous process initiated by the introduction of agriculture interspersed by periods of abandonment. We further hypothesize that this historical effect should be detectable in the present-day landscape, representing a legacy from the time before modernization of agriculture and forestry reshaped the landscape. The vast record of historical cadastral maps in Sweden provided the basis for us to test our hypothesis. Detailed maps make it possible to determine the location of different types of historical land use as well as assessing land-use change [39]. The first maps covering whole landscapes in Sweden are from around the year 1900. We can therefore only analyse the effect of land use in 1900 on the present distribution of fleshy-fruited woody species, although earlier phases of abandonment of open habitats should have led to similar legacy effects.

We asked two specific questions: (i) Is the number of fleshy-fruited species in the present landscape related to historically open and semi-open habitats? (ii) Is there a higher probability of finding fleshy-fruited woody species in areas where there has been a transition from open habitats to forest, compared to areas which were covered with forest in the past and are still forested today? In order to exclude effects of present-day open habitats, we restrict these analyses to areas with present forest cover.

## Material and methods

### Study regions

**Fig 1:**
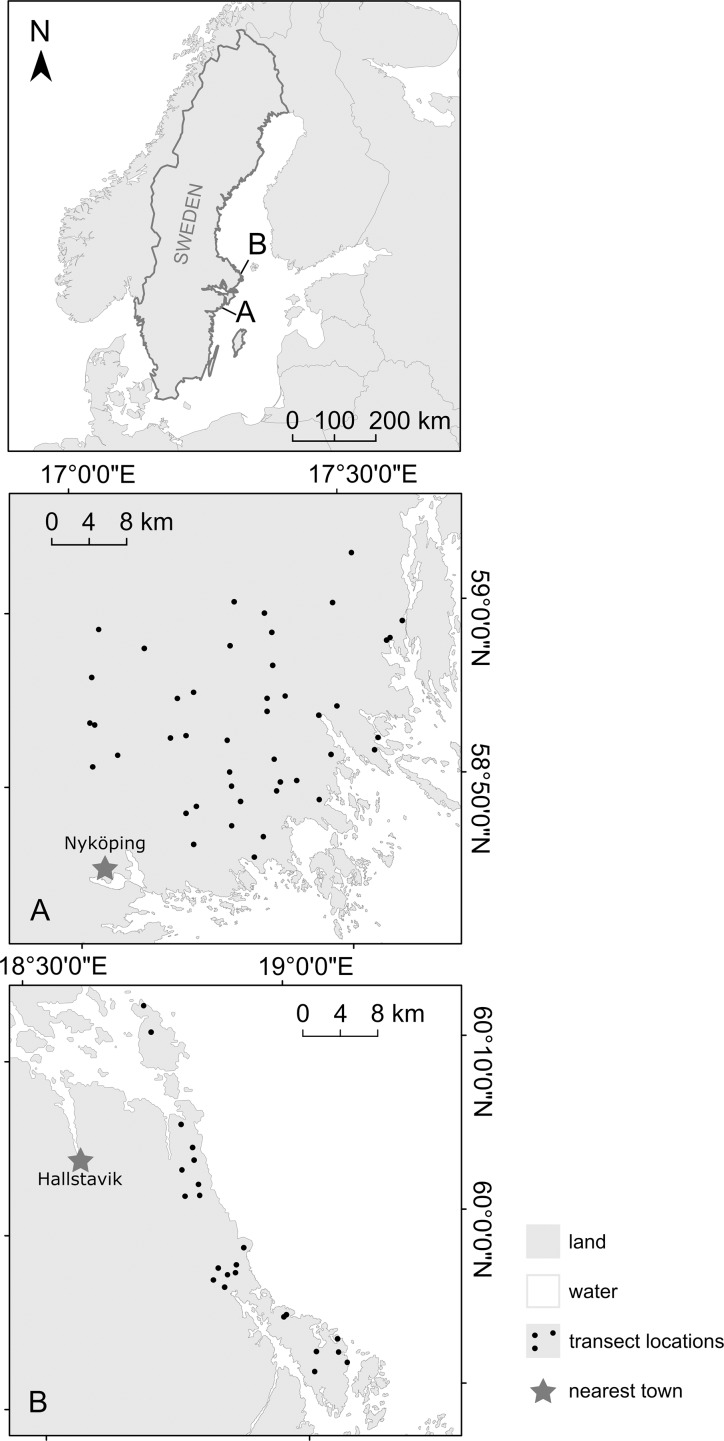
The two study regions in south-eastern Sweden. (A) Part of the County of Södermanland. (B) The peninsula of Väddö and Björkö and the island of Singö situated in the northern part of Stockholm archipelago in the Baltic Sea. The coastline of the two study regions are re-drawn from the Swedish terrain map (Terrängkartan) under a CC BY license, with permission from Lantmäteriet.

This study was conducted in two regions in southeastern Sweden (Fig 1, Table 1). Region A is located in the County of Södermanland. Region B is located in the northern part of Stockholm archipelago on the peninsula of Väddö and Björkö and the island of Singö.

**Table 1:** Description of the study regions in south-eastern Sweden. The climate data are from one climate station per region: Trosa (region A) and Singö (region B). Means are calculated for the World Meteorological Organization normal period 1961–1990 [40].

	region A	region B
County	Södermanland	Stockholm
Coordinates, midpoint (latitude/longitude)	58°54ʹ/17°19ʹ	59°58ʹ/18°51ʹ
Mean annual percipitation (mm)	559	595
Mean temp. January (°C)	-3.1	-3.5
Mean temp. June (°C)	16.3	15.9
Approximate area (km^2^)	865	164

Generally, modernization of agriculture in Sweden occurred between the mid-19^th^ century and mid-20^th^ century. At this time, cultivation of fodder on arable fields greatly reduced the areas needed for meadows, and forest grazing was abandoned [41]. In southeastern Sweden, over 90% of the semi-natural grasslands have disappeared since the late 19^th^ century [39]. The area of arable fields in Sweden peaked in the 1920s [42] and from the 1950s many fields were planted with trees, predominantly spruce.

In region A, land managed by large estates was associated with an early transition to modern agriculture, turning many semi-natural meadows on dryer soils into arable fields from the mid-19^th^-century [39,43]. In marginal agricultural regions like Stockholm archipelago, modernization of agricultural practices occurred comparatively late, and meadows and wooded meadows were to some extent still in use until the 1950s [44]. Historically, in the Stockholm archipelago, leaf hay collected from wooded meadows was particularly important [35].

The soils in both regions, apart from subsequent peat formation, were deposited after the last glaciation. Soil deposition follows the topography, with bare bedrock on hilltops and different types of glacial moraine, silt and clay in lower parts of the landscape. Region A contains larger continuous areas with soil textures suitable for crop production compared to region B where silt and clay are mostly confined to small depressions in the landscape.

### Map data

**Fig 2:**
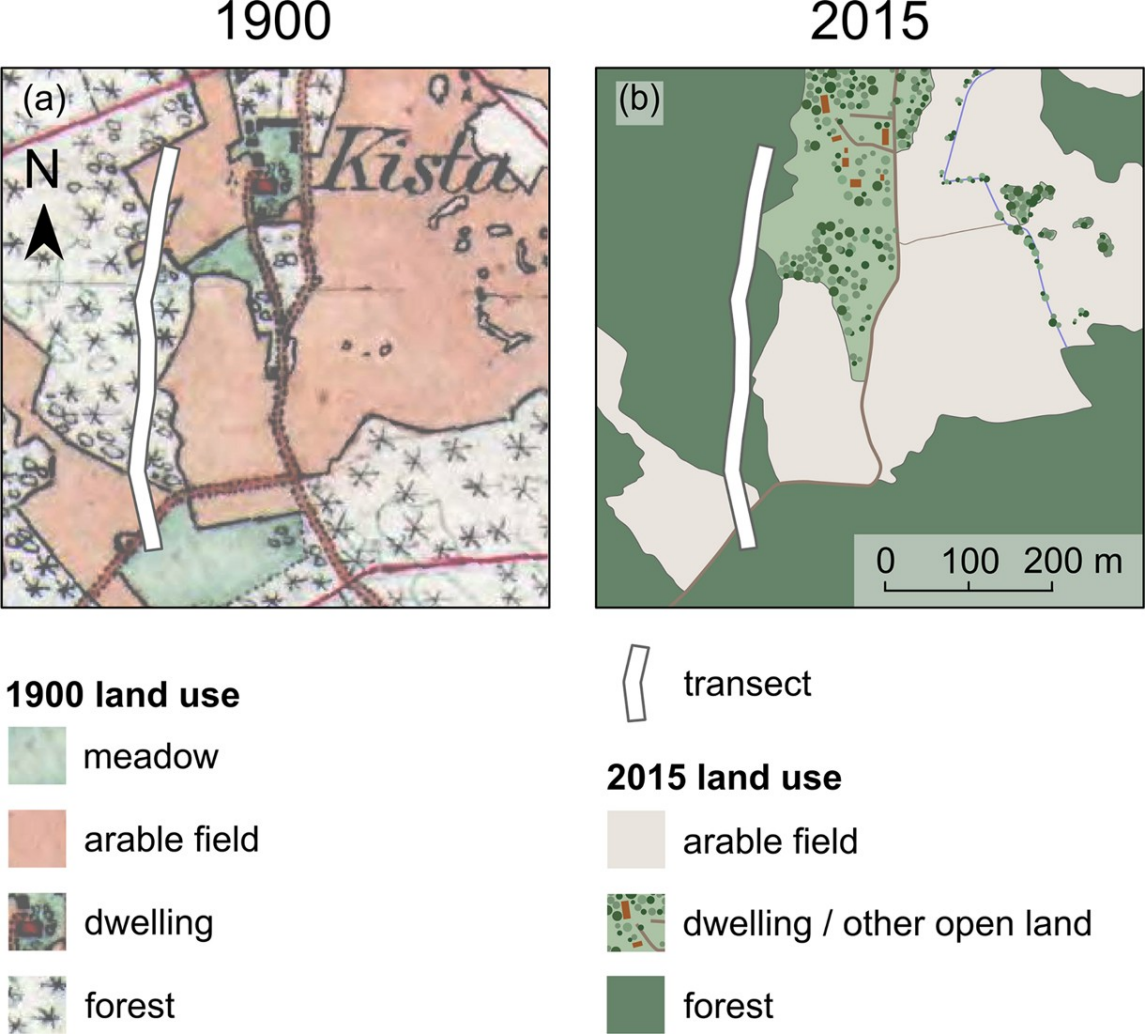
Past and present land use in Väddö-Björkö-Singö (region B). (a) Land use in 1900 from the District Economic map (in Swedish: Häradsekonomiska kartan). (b) Illustration of present land use. The District Economic map is re-printed under a CC BY license, with permission from Lantmäteriet. The illustration of present land use is drawn from an orthoimage over the study area provided by Lantmäteriet.

In order to analyse land-use change, cadastral maps were used. The first cadastral maps covering whole landscapes in Sweden are the District Economic maps (in Swedish: Häradsekonomiska kartan), at a scale of 1:50 000. The District Economic map was produced for taxation of production on arable fields and meadows, thus the delineation of open habitats is very accurate considering the age of the map. For Södermanland (region A) these maps are from 1897–1901, and for Väddö-Björkö-Singö (region B) the maps are from 1901–1906. For simplicity, we henceforth refer to these maps as being from 1900. Fig 2A shows the District Economic Map for one of the studied landscapes in region B. The cadastral maps from Södermanland were digitized in a previous study [39]. The cadastral maps from Väddö-Björkö-Singö were digitized for this study using ArcGIS 10 (ESRI, Redlands CA, USA). The rectification and digitization followed the same procedures as in Ref. 39.

Present-day digital land-cover data was obtained from the 2015 Swedish Terrain Map (in Swedish: Terrängkartan). All inhabited areas were classified as dwellings to be comparable with the land-use categories on the 1900 map.

We assessed land-use change within a 2000 m buffer zone around each transect. The combined area of the 2000 m buffers in region A was 471 km^2^. In region B, the combined area was 183 km^2^. Land-use categories extracted from the 1900 maps constituting open habitats were meadows, arable fields, pastures and dwellings. The remaining categories were forest, midfield islets, wetlands and open water. Present-day open habitat categories were arable fields, dwellings and ‘other open land’. The category ‘other open land’ includes remaining semi-natural grassland as well as pastures on former arable fields. Present-day forest categories included deciduous, mixed and coniferous forest. Using overlay-analysis in ArcGIS 10 (ESRI, Redlands CA, USA) we estimated to what extent open habitats in 1900 had transitioned to forest.

In 1900, 28% of region A was covered by open habitats, 7% meadow and 89% arable fields. The remaining 4% were dwellings including a small fraction of pastures. In 2015, c. 15% of open habitats had been transformed into forest. In Region B, 22% of the study area was covered by open habitats in 1900, 14% meadow and 78% arable fields. The remaining 8% were dwellings including a small fraction of pastures. In 2015, 30% of open habitats in 1900 had been transformed into forest. Thus, the overall changes in the relationship open habitats and forest during the 20^th^ century were somewhat larger in region B than in region A.

### Field data

Between June and September 2015, the occurrences of fleshy-fruited woody species was inventoried along approximately 500 m long and 20 m wide transects; 44 transects in region A and 23 in region B. The digitized maps covered a larger area in Södermanland than on Väddö-Björkö-Singö, hence the larger number of transects in region A. The 67 transects were randomly distributed on former meadows or former forests, based on the historical cadastral maps. Each transect was laid out in one of the cardinal directions (north, west, east or south) where it crossed the highest number of different types of former land use. For three genera where species identification may be difficult, records were lumped into *Cotoneaster* spp., *Crataegus* spp. and *Rosa* spp., and each of them was counted as one taxon. *Rubus idaeus* (raspberry) was identified, but all blackberry species were lumped into *Rubus fruticosus* agg., and counted as one taxon.

Surrounding estimated tree cover was recorded for every individual. This information was used to assess present forest cover. An estimated surrounding forest cover of > 30% was counted as present forest cover. In addition, the following data was collected: height of individuals, whether they had flowers or fruits and signs of browsing by wild ungulates. Geographical position were obtained for each recorded individual using a GPS.

### Analysis

In the following analyses only the parts of each transects with present forest cover were analysed. This was done in order to exclude effects of present-day open habitats.

To analyse whether historical land use in 1900 had an effect on the number of species per transect, we grouped transects into ‘containing open habitats’ in 1900, in contrast to ‘not containing open habitats’ in 1900. Present forest cover was extracted from the land-cover maps. In region A, 43 out of 44 transects had present forest cover. In region B, all 23 transects had present forest cover. The effect of presence of open habitat in 1900 on the number of species per transect was fitted with GLMs for quasi-Poisson distributions since the data showed overdispersion. Models were fitted each region separately. To account for variation in size of the area covered with forest per transect, size was included as an offset. R^2^ was calculated using to the method proposed by Nagelkerke [45,46].

Species for which there was sufficient data were analysed individually to test whether they had a higher probability of occurrence in areas where there had been a transition from open habitats to forest. In order to obtain presence-absence data, each transect was divided into 20 × 20 m segments, resulting in approximately 25 segments per transect. For each species, segments with one or more individuals were categorized as ‘present’ and all other segments as ‘absent’. Only species present in more than 30 transect segments were included in the analysis. In order to increase detail and accuracy, we used field data to assign present forest cover to each transect segment. Transects segments where one or more recorded individual had an estimated surrounding forest cover of > 30% were counted as having a present forest cover. With this definition, 54% of the transect segments (577 out of 1077) had a present forest cover in region A. In region B, 70% of the transect segments (369 out of 526) had a present forest cover. The fraction open habitat in 1900 was calculated per segment. For each species a model including the variable ‘fraction open habitat in 1900’ was fitted. This allowed us to estimate if historical land use was associated with the distribution of each species. The effect of the fraction of open habitat in 1900 (fixed effect) on the occurrence of each species was fitted with generalized linear mixed models (GLMMs) for binomial distributions. To account for variation in the effect of historical land use among transects, we included transect as random effect. The proportion of variance explained by the fixed effect (marginal R^2^) as well as the proportion of variance explained by the fixed and random effect (conditional R^2^) was calculated using the method proposed by Nakagawa & Schielzeth (2013)[46].

All statistical analyses were made in the R Language and Environment for Statistical Computing, v. 3.1.3 [47] using the stats package for fitting GLMs, the lme4 package [48] for fitting GLMMs and the piecewiseSEM package [49] for computing R^2^.

## Results

In all, 4963 individuals from 29 species of fleshy-fruited woody plants were recorded during the surveys (S1 Table). *Sorbus aucuparia* was the most common species in both regions, whereas the abundance ranking below *S*. *aucuparia* differed between the study regions. Despite a higher number of transects in region A than in region B (44 vs. 23), *Lonicera xylosteum*, *Prunus padus* and *Viburnum opulus* were more abundant in region B. *Daphne mezerum*, *Hippophaë rhamnoides*, *Rhamnus cathartica*, *Rubus fruticosus* agg., and *Sorbus hybrida* were only recorded in region B. *Rubus ideaus* and *Prunus avium* were more abundant in region A. *Amelanchier spicata*, *Prunus domestica*, *Prunus spinosa*, *Sambucus nigra* and *Sambucus racemosa* were only recorded in region A.

The number of species per transect was higher in region B compared to region A (Table 2) and the additional information gathered during surveys suggested that the population structure differed between the study regions. In region B, the fraction small individuals (< = 50cm) was lower and the fraction individuals with flowers or fruits was higher. In addition, the fraction individuals with signs of browsing by wild ungulates was lower in region B compared to region A (Table 2).

**Table 2:** Overview of occurrences and status of individual plants in the two regions.

	region A	region B
occurrences	2832	2131
species per transect	7±3.3	10±3.5
individuals ≤ 50cm	57%	36%
fertile individuals	9%	24%
signs of browsing	41%	12%

In region A, 44 transects were inventoried compared to 23 transects in region B.

Historical land use has an effect on the number of species in the landscape. Analysing parts of transects with present forest cover, the number of species was significantly higher on transects with open habitat in 1900 compared to transects without past open habitats (Table 3). When analysing each region separately, the presence of open habitats in the year 1900 was not related to the number of species per transect in region A (Table 3). This was in contrast to region B, where the number of species per transect was significantly positively related to the occurrence of open habitats in the year 1900 (Table 3).

**Table 3:** Effect of historical land use on the number of fleshy-fruited woody species per transect in today´s forest.

			Estim.	SE	t-value	p-value	Sign.	R^2^
**Region A**								
	open habitat in 1900		0.464	0.325	1.426	0.161		0.08
**Region B**								
	open habitat in 1900		0.574	0.229	2.509	0.020	*	0.26

Open habitats in 1900: meadows, pastures, arable fields and dwellings.

* Significant at p < 0.05

** significant at p < 0.005

*** significant at p < 0.001.

**Fig 3:**
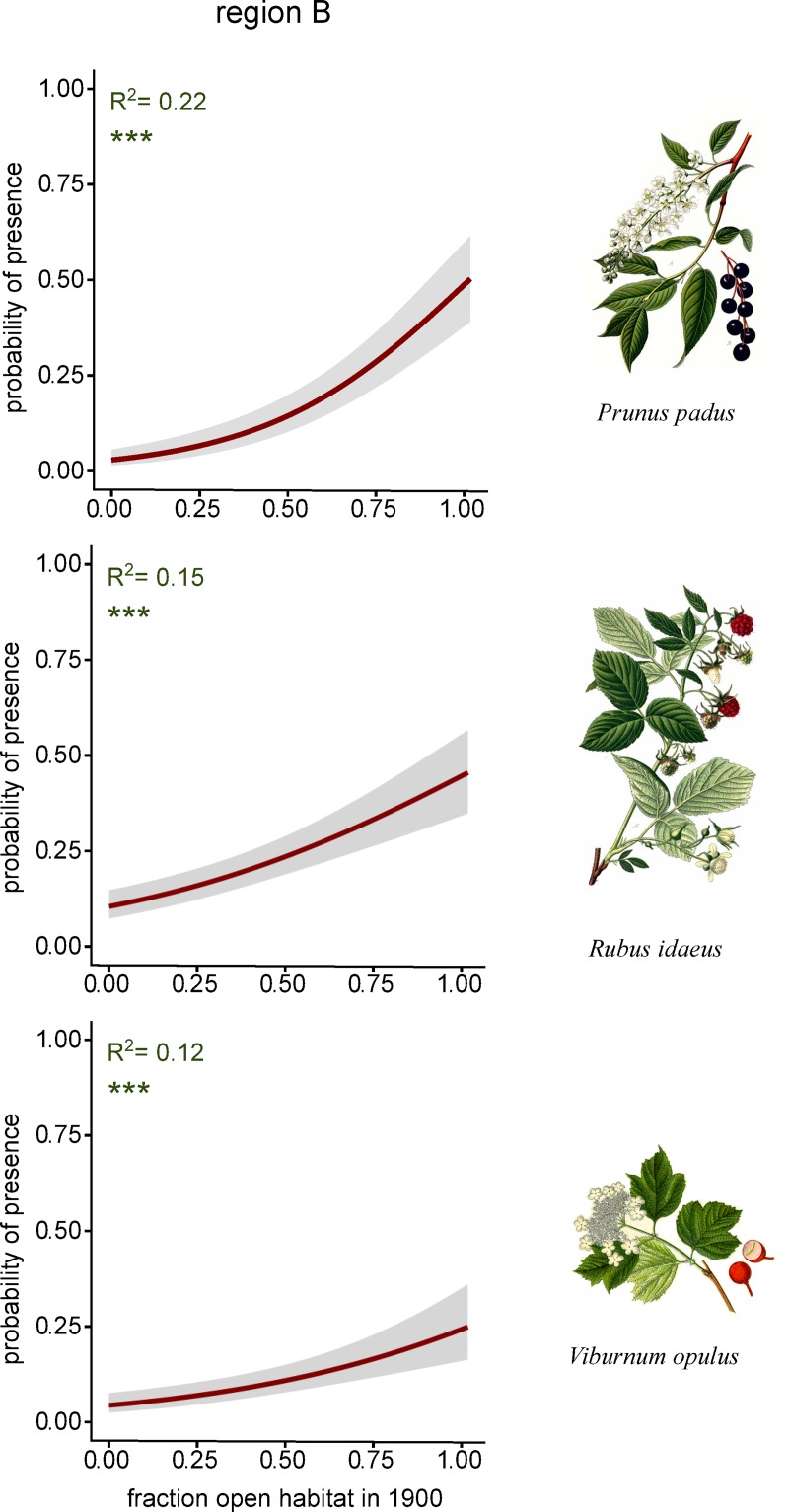
Effect of historical land use on the occurrence of fleshy-fruited woody in today’s forest. Results from testing the effect of fraction open habitat in 1900 on the occurrence of individual species in the two study regions. The predicted probabilities are based on the intercept of the fixed effect. The results are visualized for the three species where models showed the highest explanatory power. The drawings of *Prunus padus* and *Rubus idaeus* are redrawn from the Atlas des plantes de France (Masclef 1891) which is part of the public domain. The drawing of *Viburnum opulus* is redrawn from the Deutschlands Flora in Abbildungen (Sturm 1796) which is part of the public domain.

Table 4 presents the results of how a transition from historically open habitats in 1900 to present-day forest affects the occurrence of fleshy-fruited woody species today. In region B, five out of nine examined species showed a historical signal in their distribution patterns, such that their present-day occurrence was positively related to historical land use in 1900. *L*. *xylosteum*, *P*. *padus*, *R*. *alpinum*, *R*. *idaeus* and *V*. *opulus* had a higher probability of occurring in parts of transects with a large fraction open habitats in 1900. The explanatory power (R^2^) of the models ranged from 0.03 to 0.22. In region A, two out of nine examined species showed a historical signal, such that their present-day occurrence was positively related to historical land use in 1900 (Table 4). *Rubus idaeus* and *Rosa spp*. had a higher probability of occurrence in parts of transects with a large proportion of open habitats in 1900. However, the explanatory power of the models was weak, 0.01–0.02. *Juniperus communis* did also show a historical signal in both regions, but in the opposite direction as compared to the species mentioned above (Table 4). In both region A and B, *J*. *communis* had a lower probability of occurrence in parts of transects with a large fraction of open habitats. In Fig 3 the results are visualized for the three species where models showed the highest explanatory power: *P*. *padus* (region B, R^2^ = 0.22), *R*. *idaeus* (region B, R^2^ = 0.15), *V*. *opulus* (region B, R^2^ = 0.12).

**Table 4:** Effect of historical land use on the occurrence of fleshy-fruited woody species in today’s forest.

			N	Estim.	SE	z-value	p-value	Sign.	R^2^_(m)_	R^2^_(c)_
**region A**										
	fraction open habitat in 1900									
		*Frangula alnus*	68	0.738	0.443	1.666	0.096		0.01	0.46
		*Juniperus communis*	195	-1.743	0.411	-4.243	<0.001	***	0.07	0.44
		*Lonicera xylosteum*	22							
		*Prunus avium*	38	0.736	0.577	1.274	0.203		0.01	0.64
		*Prunus padus*	3							
		*Prunus spinosa*	31	0.601	0.620	0.969	0.333		0.01	0.63
		*Ribes alpinum*	95	-0.491	0.436	-1.126	0.260		0.00	0.55
		*Rosa spp*.	92	0.916	0.368	2.490	0.013	*	0.02	0.42
		*Rubus idaeus*	329	0.685	0.317	2.158	0.031	*	0.01	0.30
		*Sorbus aucuparia*	387	-0.389	0.350	-1.112	0.266		0.00	0.51
		*Sorbus intermedia*	62	-0.208	0.470	-0.444	0.657		0.00	0.41
		*Viburnum opulus*	1							
**region B**										
	fraction open habitat in 1900									
		*Frangula alnus*	70	-0.209	0.543	-0.385	0.700		0.00	0.34
		*Juniperus communis*	114	-1.337	0.410	-3.264	0.001	**	0.06	0.28
		*Lonicera xylosteum*	103	1.517	0.446	3.402	<0.001	***	0.04	0.60
		*Prunus avium*	2							
		*Prunus padus*	70	3.460	0.586	5.905	<0.001	***	0.22	0.62
		*Prunus spinosa*	0							
		*Ribes alpinum*	147	0.980	0.360	2.723	0.006	**	0.03	0.38
		*Rosa spp*.	54	0.593	0.419	1.417	0.157		0.01	0.17
		*Rubus idaeus*	72	1.928	0.338	5.705	<0.001	***	0.15	0.19
		*Sorbus aucuparia*	314	-0.682	0.442	-1.541	0.123		0.02	0.26
		*Sorbus intermedia*	30							
		*Viburnum opulus*	49	1.920	0.473	4.064	<0.001	***	0.12	0.34

Open habitats in 1900: were meadows, pastures, arable fields and dwellings. N: number of transects segments where the species was present. Models were only fitted for species where N > 30. R^2^_(m)_: proportion of variance explained by the fixed effect (marginal R^2^). R^2^_(c)_: proportion of variance explained by the fixed and random effect (conditional R^2^).

* Significant at p < 0.05

** significant at p < 0.005

*** significant at p < 0.001.

## Discussion

In this work, we have examined whether there is a legacy of historical land use affecting the occurrence, distribution and species richness of fleshy-fruited woody species in the present-day landscape. In a study of occurrences of fleshy-fruited species, van Ruremonde & Kalkhoven (1991) found that effects of woodlot isolation on *Lonicera periclymenum* partly had a historical component [50]. However, apart from that study, to our knowledge, no other comparable study has been conducted on fleshy-fruited woody species, or with a focus on an ecological guild rather than on all plants in a specific habitat. The hypothesis was based on a combination of effects, the human-mediated changes in landscape structure [15], the response of frugivores to such changes [23–25] and the intentional favouring of these species in managed habitats [26]. Furthermore, the predicted accumulation of species should be detectable at a landscape scale, and not just in single grasslands, where such historical effects on grassland species have been documented in several studies [11,12,19,22]. In order to investigate historical effects on landscape scale species richness of fleshy-fruited woody plants we needed historical maps covering relatively large areas (which constrained us to maps from around the year 1900).

In congruence with our hypothesis, we found an overall effect of historical land use on species richness of fleshy-fruited woody species. By analysing species richness in present-day forests, we excluded the potential effects of present open habitats. We found the number of species to be significantly higher in transects with open habitat in 1900 compared to transects without open habitats in the past. When analysing the regions separately this effect was significant in Väddö-Björkö-Singö (region B) located in the northern part of Stockholm archipelago, but not in the county of Södermanland (region A). However, patterns of species richness primarily depend on processes affecting species individually. Accordingly, we analysed how a transition from open habitats in 1900 to forest today affected species occurrences, as compared to sites where forest existed already in the year 1900.

In line with the patterns of species richness, the strongest support for our hypothesis was found in region B. Out of nine species that could be analysed, five species (*L*. *xylosteum*, *P*. *padus*, *R*. *alpinum*, *R*. *ideaus* and *V*. *opulus*) are more likely to occur at sites where there has been a transition from open habitats to forest. Thus, variation in the occurrences of these species in present-day forests partly reflects different ecological and geographical conditions historically. Overall, between 3% and 22% of the variation in species occurrences was statistically explained by historical land use. While this suggests that other factors than historical land use are also important, these figures are in the same magnitude as results obtained in a study of temperate forest understory plants [51], which estimated that historical factors accounted for 13% of the variation in species composition. In contrast to region B, we found only very weak historical effects in region A. In this region, only two species were more likely to occur at sites where there has been a transition from open habitats to forest, and the effects was weak (1–2% of the variation in these species’ occurrences was statistically explained by historical land use alone).

What may be the reason for this difference between regions? The area of agricultural fields in Sweden expanded from the late 19^th^ century and reached its peak in the 1920s [42] and from the 1950s many arable fields were planted with trees, predominantly spruce. Our land-use change analysis reviles that in region B, a larger proportion of open habitats in the year 1900 compared to region A, was later transformed into forest. Thus, a larger proportion of the present forest is of younger date in region B. Accordingly, if there is an effect of historical land use (as evident in region B), and this effect is declining over time, it would explain why we did detected a historical effect in region B and no (or just a very weak effect) in region A. In region B, old management forms such as hay-making on meadows were retained later than in in region A and the intentional favouring of fleshy fruited species may consequently also have been practiced later in this region. If this interpretation is correct, our results suggest that the time-scale for the disappearance of historical effects on the distribution of fleshy-fruited woody plants is in the order of magnitude of a century, similar to what has been found in grasslands [12].

An additional factor which may influence the difference between the regions is browsing by large wild herbivores, such as moose, fallow deer and roe deer. Browsing is more common in region A, where the fraction of individuals with signs of browsing was more than twice as high region B. As the abundance of these wild herbivores have increased in Sweden over the last century [52], it may act to speed up the disappearance of the historical signal in some regions.

In both regions, *J*. *communis* was the only species showing a negative effect to historically open habitats. *Juniperus communis* may have been actively favoured by humans because of its edible cones and wood suitable for carpentry. However, *J*. *communis* may also have been actively removed from meadows due to the negative impact of litter from coniferous species. This would explain its negative association with past open habitats. Moreover, since its needles offer a natural protection against browsing, *J*. *communis* may have been more common outside the enclosed open areas.[53,54].

The most common species in our study, *S*. *aucuparia*, did not show any historical signal in its distribution. A landscape study of regeneration in *S*. *aucuparia* found that despite a clumped distribution of adults, there was a high density of seedlings and saplings, even in dense spruce stands [55]. Thus, *S*. *aucuparia* is likely capable of establishing in most habitats, and therefore any effect of historical land use (e.g. if *S*. *aucuparia* was intentionally favoured on infields) is undetectable.

In contrast to most previous studies on historical effects (but see reference 49), where studies have concerned specific geographical areas or vegetation types, we focused on a guild of species sharing a specific seed dispersal system. Seed dispersal of fleshy-fruited woody species is mainly executed by frugivores, in our study region mostly birds. Fruits provide an important food source for many bird species, and the availability of fleshy fruits influences the abundance of frugivorous birds [56]. Thus, to the extent that there are historical effects on fleshy-fruited woody species, these effects may cascade to abundance and distribution of birds. As the foraging by birds also feedbacks to the dispersal and recruitment of plants [32], this implies that the historical effects in fact may influence a complex network of plant-frugivore interactions [57].

Our results can be placed in a wider context, both geographically and ecologically. It has been recognized that many useful fleshy-fruited plants are typically occurring in wood pastures across Europe, and were most likely intentionally favoured [10]. Biological legacies of previous management, mainly forest grazing, are abundant in boreal and nemo-boreal forests and concern also other plants than woody species with fleshy fruits as well as lichens, insects and birds [58].

In conclusion, we suggest that historical land use affects the occurrence and distribution of several fleshy-fruited woody species, and that these patterns are manifested at a landscape scale. Our study was limited to two study regions, due to the constraints of digitizing historical maps covering large areas. Historical anthropogenic effects in vegetation have probably always existed, ever since humans started to transform landscapes. These effects are manifested as a historical signal. Based on a comparison between these two regions, we suggest that the historical signal regarding fleshy-fruited woody plants in former infield systems may be detectable for approximately one century. We believe that these conclusions deserve to be the focus of coming studies.

## Supporting information

S1 Fig Historical presence of woody species in wooded meadows on islands in Åland archipelago in the Baltic Sea (Palmgren 1916, Ref. 34). Gray bars indicate fleshy-fruited species. Eighteen out of 28 fleshy-fruited species were present in more than 50% of the wooded meadows (indicated by the dashed line). (*) species that have changed scientific name since Palmgren’s inventory. New scientific names: *Alnus glutinosa* (L.) Gaertner (*Alnus rotundifolia*), *Malus sylvestris* Mill. (*Pyrus malus*), *Sorbus hybrida* L. (*Sorbus fennica*), *Betula pendula* Roth (*Betula verrucosa*), *Frangula alnus* Mill. (*Rhamnus frangula*), *Sorbus intermedia* (Ehrh.) Pers. (*Sorbus suecica*), *Crataegus monogyna* Jacq. (*Mespilus monogyna*), *Ulmus glabra* Huds. (*Ulmus scabra*). (**) *Rosa* L. species that may have changed scientific name since Palmgren’s inventory. Some *Rosa* species share scientific synonyms, wherefore it is difficult to know with certainty which species Palmgren (1916) was referring to. Ref. 34: Palmgren A. Studier öfver löfängsområdena på Åland. III Statistisk undersökning af floran. Acta Soc Fauna Flora Fenn. 1916;42: 479–633.
(PDF)

S1 Table Number of occurrences of fleshy-fruited woody species in the two study regions. In region A, 44 transects were inventoried. In region B, 23 transects were inventoried.
(PDF)
