# Supplementary material for: Does historical land use affect the regional distribution of fleshy-fruited woody plants?
Source: PLoS One. 2019 Dec 5;14(12):e0225791. doi: 10.1371/journal.pone.0225791 (PMC6894828; doi:10.1371/journal.pone.0225791)
Supplement: S1 Fig — Gray bars indicate fleshy-fruited species. Eighteen out of 28 fleshy-fruited species were present in more than 50% of the wooded meadows (indicated by the dashed line). (*) species that have changed scientific name since Palmgren’s inventory. New scientific names: Alnus glutinosa (L.) Gaertner (Alnus rotundifolia), Malus sylvestris Mill. (Pyrus malus), Sorbus hybrida L. (Sorbus fennica), Betula pendula Roth (Betula verrucosa), Frangula alnus Mill. (Rhamnus frangula), Sorbus intermedia (Ehrh.) Pers. (Sorbus suecica), Crataegus monogyna Jacq. (Mespilus monogyna), Ulmus glabra Huds. (Ulmus scabra). (**) Rosa L. species that may have changed scientific name since Palmgren’s inventory. Some Rosa species share scientific synonyms, wherefore it is difficult to know with certainty which species Palmgren (1916) was referring to. Ref. 34: Palmgren A. Studier öfver löfängsområdena på Åland. III Statistisk undersökning af floran. Acta Soc Fauna Flora Fenn. 1916;42: 479–633. (PDF) [file pone.0225791.s001.pdf]

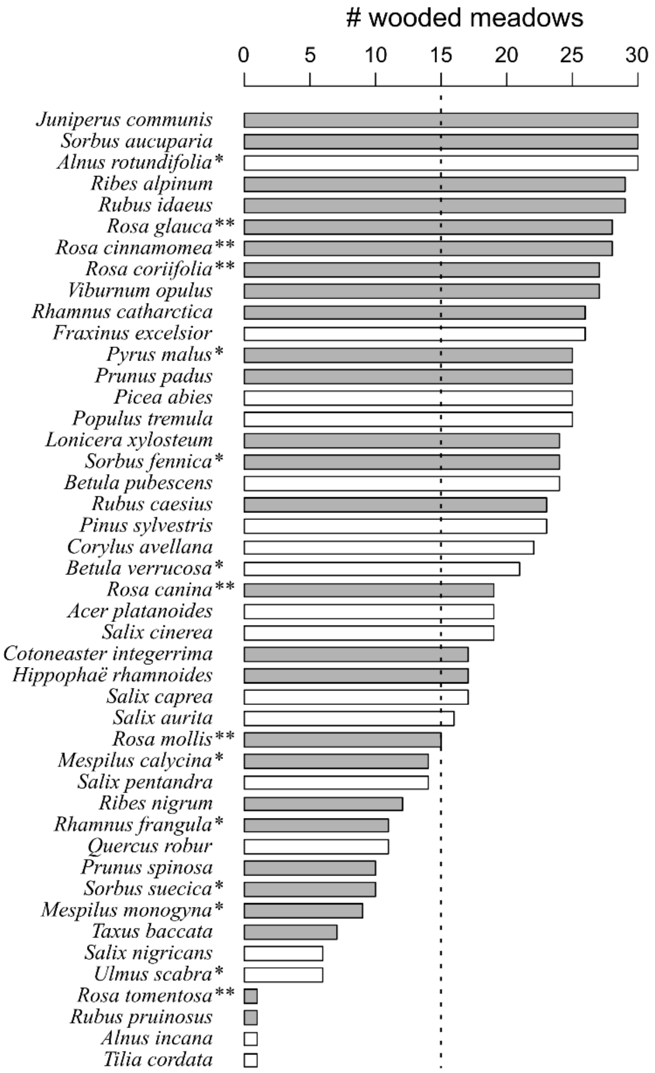

S1 fig. **Historical presence of woody species in wooded meadows on islands in Åland archipelago in the Baltic Sea (Ref. 34: Palmgren 1916).** Gray bars indicate fleshy-fruited species. Eighteen out of 27 fleshy-fruited species were present in more than 50% of the wooded meadows (indicated by the dashed line). (*) species that have changed scientific name since Palmgren's inventory. New scientific names: *Alnus glutinosa* (L.) Gaertner (*Alnus rotundifolia*), *Malus sylvestris* Mill. (*Pyrus malus*), *Sorbus hybrida* L. (*Sorbus fennica*), *Betula pendula* Roth (*Betula verrucosa*), *Frangula alnus* Mill. (*Rhamnus frangula*), *Sorbus intermedia* (Ehrh.) Pers. (*Sorbus suecica*), *Crataegus monogyna* Jacq. (*Mespilus monogyna*), *Ulmus glabra* Huds. (*Ulmus scabra*). (**) *Rosa* L. species that may have changed scientific name since Palmgren's inventory. Some *Rosa* species share scientific synonyms, wherefore it is difficult to know with certainty which species Palmgren (1916) was referring to. Ref. 34: Palmgren A. Studier öfver löfängsområdena på Åland. III Statistisk undersökning af floran. Acta Soc Fauna Flora Fenn. 1916;42: 479–633.
