# Supplementary material for: Does historical land use affect the regional distribution of fleshy-fruited woody plants?
Source: PLoS One. 2019 Dec 5;14(12):e0225791. doi: 10.1371/journal.pone.0225791 (PMC6894828; doi:10.1371/journal.pone.0225791)
Supplement: S1 Table — In region A, 44 transects were inventoried. In region B, 23 transects were inventoried. (PDF) [file pone.0225791.s002.pdf]

S1 table. **Number of occurrences of fleshy-fruited woody species in the two study regions.** In region A, 44 transects were inventoried. In region B, 23 transects were inventoried.

	**region A**	**region B**
Sorbus aucuparia	873	674
Rubus idaeus	762	162
Juniperus communis	373	211
Rosa spp.	181	120
Ribes alpinum	172	289
Frangula alnus	112	117
Sorbus intermedia	71	40
Prunus avium	69	2
Prunus spinosa	64	0
Lonicera xylosteum	41	203
Crataegus spp.	19	6
Ribes uva-crispa	18	8
Malus sylvestris	16	15
Sambucus racemosa	13	0
Ribes spicatum	12	12
Amelanchier spicata	9	0
Berberis vulgaris	7	2
Prunus padus	7	137
Sambucus nigra	5	0
Ribes nigrum	3	5
Prunus domestica	2	0
Cotoneaster spp.	1	3
Malus domestica	1	3
Viburnum opulus	1	72
Daphne mezereum	0	32
Hippophaë rhamnoides	0	3
Rhamnus cathartica	0	13
Rubus fruticosus agg.	0	1
Sorbus hybrida	0	1
